# Maternal body mass index in early pregnancy and severe asphyxia-related complications in preterm infants

**DOI:** 10.1093/ije/dyaa088

**Published:** 2020-06-26

**Authors:** Ayoub Mitha, Ruoqing Chen, Stefan Johansson, Neda Razaz, Sven Cnattingius

**Affiliations:** 1 Division of Clinical Epidemiology, Department of Medicine Solna, Karolinska Institutet, Stockholm, Sweden; 2 CHU Lille, Department of Neonatal Medicine, Jeanne de Flandre Hospital, Lille, France; 3 Université de Paris, Epidemiology and Statistics Research Center/CRESS, INSERM (U1153-Obstetrical, perinatal and Pediatric Epidemiology Research Team (EPOPé)), INRA, Hôpital Tenon, Bâtiment Recherche, Rue de la Chine, Paris, France; 4 Department of Clinical Science and Education, Södersjukhuset, Stockholm, Sweden

**Keywords:** Maternal body mass index, preterm morbidity, nationwide cohort study

## Abstract

**Background:**

Little is known about the associations between maternal body mass index (BMI) and asphyxia-related morbidity in preterm infants (<37 weeks). We aimed to investigate associations between maternal BMI in early pregnancy and severe asphyxia-related neonatal complications in preterm infants (<37 weeks) and to examine whether possible associations were mediated by overweight- or obesity-related complications.

**Methods:**

In this Swedish population-based cohort of 62 499 singleton non-malformed preterm infants born from 1997 to 2011, risks of low Apgar scores (0–3) at 5 and 10 minutes, neonatal seizures and intraventricular haemorrhage (IVH) were estimated through two analytical approaches. In the conventional approach, the denominator for risk was all live births at a given gestational age. In the fetuses-at-risk (FAR) approach, the denominator for risk was ongoing pregnancies at a given gestational age.

**Results:**

Using the conventional approach, adjusted risk ratios per 10-unit BMI increase were 1.32 [95% confidence interval (CI) 1.13–1.54] and 1.37 (95% CI 1.12–1.67) for low Apgar scores at 5 and 10 minutes, respectively; 1.28 (95% CI 1.00–1.65) for neonatal seizures; and 1.18 (95% CI 1.01–1.37) for IVH. Using the FAR approach, corresponding risks were higher. These associations varied by gestational age (<32 and 32–36 weeks). Associations between maternal BMI and asphyxia-related outcomes were partly mediated through lower gestational age.

**Conclusions:**

Increasing maternal BMI in early pregnancy is associated with increased risks of severe asphyxia-related complications in preterm infants. Our findings add to the evidence to support interventions to reduce obesity in woman of reproductive age.

**Key Messages**

- Little is known about associations between maternal body mass index (BMI) and asphyxia-related morbidity in preterm infants.
- Increasing maternal BMI in early pregnancy is associated with increased risks of low Apgar scores at 5 and 10 minutes, neonatal seizures and intraventricular haemorrhage in preterm infants.
- Associations between maternal BMI and asphyxia-related outcomes are partly mediated through lower gestational age.
- Our findings add to the evidence to support interventions to reduce obesity in woman of reproductive age.

## Introduction

The prevalence of overweight and obesity among pregnant woman has increased worldwide.^1^ From 1992 to 2010, the prevalence of overweight and obesity [body mass index (BMI) ≥25] in early pregnancy increased from 26% to 38% in Sweden.^2^^,^^3^ Women’s weight before and during pregnancy affects the course and outcome of pregnancy, as well as offspring health.^1^ In term infants (≥37 weeks), maternal overweight (BMI 25 to <30) and obesity (BMI ≥30) are associated with increased risks of neonatal mortality and morbidity.^1^ In term infants, risks of severe asphyxia-related complications, such as low (0–3) Apgar scores at 5 and 10 minutes, neonatal seizures and asphyxia-related infant mortality, increase with maternal BMI in a dose–response manner.^2^^,^^4^ However, few studies have examined the associations between maternal BMI and neonatal morbidity in preterm (<37 weeks) infants.^1^^,^^2^

Maternal overweight and obesity during pregnancy can be regarded as a risk factor for preterm delivery, particularly for extremely preterm (22–27 weeks) delivery.^5^ Decreasing gestational age, as well as hypertensive and diabetic diseases that may be related to high maternal BMI, have also been associated with a higher risk of neonatal morbidity.^1^^,^^6–11^ However, the existing literature has, to the best of our knowledge, never explored the possible underlying pathways for these associations.

In this nationwide Swedish cohort study, we aimed to investigate the associations between maternal BMI in early pregnancy and risks of severe asphyxia-related neonatal complications in preterm infants (<37 weeks); and in very (<32 weeks) and in moderately (32–36 weeks) preterm infants, respectively. We also aimed to examine whether these associations were mediated by overweight- or obesity-related pregnancy and delivery complications.

## Methods

### Data sources

This nationwide cohort study was based on data from the Swedish Medical Birth Register, which includes data on >98% of all births in Sweden.^12^ The validity of most variables included in the Medical Birth Register is considered high.^12^ Using the unique personal identity number assigned to all Sweden residents,^13^ we linked data from the Medical Birth Register with the National Patient Register,^14^ Cause of Death Register,^15^ Total Population Register^16^ and Education Register.^17^

The Medical Birth Register includes prospectively collected standardized information from prenatal, obstetric and neonatal records. The National Patient Register includes information on diagnoses and dates on hospital-based in-patient care, and diagnoses have since 1997 been coded according to the Swedish version of the International Classification of Diseases, Tenth Revision (ICD 10-codes). Information on the date and cause of death was derived from the Cause of Death Register and information on the mother’s country of birth and level of education was retrieved from the Total Population Register and Education Register, respectively.

The study was approved by the Regional Ethical Review Board in Stockholm, Sweden (No. 2012/1813–31/4).

### Study population

Between 1997 and 2011, the Medical Birth Register included information on 1 490 817 births. We excluded term births (i.e. gestational age ≥37 weeks) (*n* = 1 395 348), stillbirths (*n* = 2669), births with missing data on gestational age (*n* = 1299) and Apgar score at 5 minutes (*n* = 1196), incomplete maternal or infant personal identity numbers (*n* = 1908), multiple births (*n* = 19 229) and infants with major congenital malformation (*n* = 6669). Our study included 62 499 singleton live-born non-malformed preterm infants. Information about the ICD 10-codes for major congenital malformations is provided in Supplementary Table 1, available as Supplementary data at *IJE* online.

### Exposure

Maternal weight was measured in light indoor clothes and maternal height was self-reported at the first antenatal visit, occurring within the first 12 weeks of gestation in 90% of all pregnancies.^12^ Maternal BMI (weight in kilograms divided by the square of the height in metres) was categorized according to the World Health Organization definition of underweight (<18.5), normal weight (18.5–24.9), overweight (25–29.9) and obesity grades I (30–34.9), II (35–39.9) and III (≥40). Because the numbers of women in the BMI groups <18.5 and ≥40 were small, we used <25 and ≥35 as the cut-offs for the lightest and heaviest BMI groups, respectively.

### Definition of outcomes

The outcomes of interest were severe asphyxia-related complications during the neonatal period (0–27 completed days of age). Information on Apgar scores was derived from the Medical Birth Register. Low (0–3) Apgar scores at 5 and 10 minutes were exclusive: a low Apgar score at 5 minutes was defined as an Apgar score of 0–3 at 1 and 5 minutes, and an Apgar score of 4–10 at 10 minutes. A low Apgar score at 10 minutes was defined as an Apgar score of 0–3 at 1, 5 and 10 minutes.

Other severe asphyxia-related complications included neonatal seizures (ICD-10 P90) and intraventricular haemorrhage (IVH) grades 1–4 (ICD-10 P52.0-52.2) diagnosed by head ultrasounds.^18^ Severe IVH (grades 3–4; ICD-10 P52.2) was also examined.^18^ Diagnostic information was based on ICD-codes recorded as a diagnosis (Medical Birth Register and Patient Register) or as a cause of death (Cause of Death Register).

### Confounders

Baseline characteristics that have been associated with both maternal BMI and risk of neonatal asphyxia-related complications were considered as potential confounders. Maternal characteristics included age at delivery, height, parity, smoking during pregnancy, highest education level, country of birth and year of delivery.^1^^,^^2^^,^^4^^,^^5^^,^^19^ Maternal age at delivery was defined as the date of delivery minus the mother’s birth date. Parity was the number of births of each mother (including the present birth). Smoking status was collected by self-report at the first prenatal visit and has been previously validated using cotinine markers.^20^

Gestational age was determined using the following hierarchy: early second-trimester ultrasound (83.0%), date of last menstrual period reported at the first prenatal visit (8.7%) or from a postnatal assessment (8.3%). The length of gestation was categorized as moderately (32–36 weeks) and very (<32 weeks) preterm. From the ultrasound-based sex-specific Swedish reference curve for fetal growth,^21^ we calculated the Z scores of birthweight-for-gestational age, which were converted to birthweight percentiles.

### Mediators

We considered pregnancy and delivery complications of maternal overweight and obesity as potential mediators for the association between early-pregnancy BMI and severe asphyxia-related neonatal complications, including maternal hypertension and diabetes, preterm premature rupture of the membranes, gestational age and emergency deliveries (including emergency caesarean and vaginal instrumental deliveries).^1^ Maternal hypertension and diabetes were defined as having any of the following diseases: chronic hypertension, pre-eclampsia, pre-gestational diabetes or gestational diabetes (ICD-10 codes for maternal diseases and preterm premature rupture of the membranes are shown in Supplementary Table 1, available as Supplementary data at *IJE* online).

### Statistical analysis

We first calculated the rates of low Apgar scores at 5 and 10 minutes, neonatal seizures and IVH as the proportion of infants with these outcomes per 1000 live births, according to each maternal and infant characteristic.

Poisson-regression analysis was performed to estimate the risk ratios (RRs) of severe asphyxia-related neonatal complications in relation to BMI as a continuous variable (per 10 units of BMI). Ten units was approximately the difference between the median BMIs of mothers with obesity (33.1) and without obesity (23.0). Maternal BMI has been shown to correlate with the risk of neonatal complications in a U-shaped relationship.^5^ We therefore first tested whether there was a nonlinear association between maternal BMI and risk of neonatal complications by extending the Poisson-regression model with a quadratic term. As we found no evidence of a nonlinear association (Supplementary Table 2, available as Supplementary data at *IJE* online), we examined the linear association between maternal BMI and risk of neonatal complications. The analyses were also performed in different maternal BMI categories (BMI 25 to <30, 30 to <35 and ≥35) compared with women with BMI <25. RRs and 95% confidence intervals (CIs) were adjusted for the potential confounders described above. To account for the correlation among infants with the same mother, we used a robust sandwich estimator to correct standard errors in all analyses. To assess the gestational-age-specific risk for neonatal morbidity, we also estimated the RRs separately for very preterm and moderately preterm infants, respectively. In the conventional analysis, the denominator for the rate or risk was all live preterm births in a given gestational-age interval.

**Figure 1:**
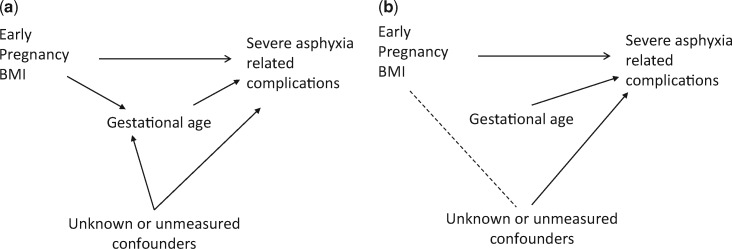
Directed acyclic graph representing the role of gestational age. (A) Without stratifying or (B) stratifying on gestational age. Stratifying on gestational age as a ‘collider’ opens the path ‘Early pregnancy body mass index (BMI)—Unknown or unmeasured confounders—Severe asphyxia related complications’ and may result in biased estimates of associations between early-pregnancy BMI and severe asphyxia-related complications. Gestational age is known as a ‘collider’, as it can be affected by both early-pregnancy BMI and unknown or unmeasured confounders in the association between gestational age and severe asphyxia-related complications.

Although the conventional approach has been widely applied in the studies of neonatal morbidity, results might suffer from collider stratification bias when stratifying by gestational age in the presence of unmeasured factors that are causes of both preterm birth and neonatal morbidity (Figure 1).^22^ Such a bias can lead to erroneous estimates and paradoxical results.^23^ The fetuses-at-risk (FAR) approach has been proposed to preclude biased paradoxical associations.^24^^,^^25^ In the FAR analysis, the denominator for the rate or risk is ongoing pregnancies at a given gestational age.^24^^,^^25^ Rates of severe asphyxia-related neonatal complications were then calculated as the proportion of infants with these outcomes per 1000 live fetuses. Therefore, using the FAR approach, we performed analysis in a larger cohort, in which we additionally included 1 295 867 term (≥37 weeks) infants. The RR estimation for the FAR approach was performed with the same analytic models as used for the conventional approach.

**Figure 2:**
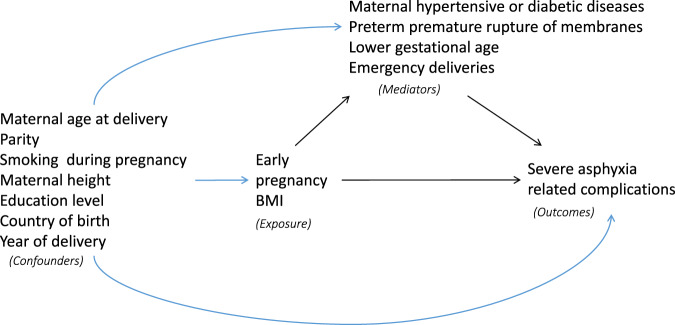
Directed acyclic graph illustrating the possible structural relationship between early-pregnancy body mass index (BMI), confounders, mediators and severe asphyxia-related complications.

To examine whether the associations between maternal BMI and severe asphyxia-related neonatal complications could be mediated by overweight or obesity-related pregnancy and delivery complications, we performed mediation analyses using the CAUSALMED procedure in SAS that implements the regression approach of VanderWeele.^26^  Figure 2 shows the exposure, mediators, outcomes and covariates that confound the relationship among exposure, outcomes and mediators in a causal diagram. We assumed that adjustment for baseline characteristics is adequate to adjust for confounding for the associations between exposure and outcome, between mediator and outcome, and between exposure and mediator. The birthweight-for-gestational age percentile was considered as a potential common risk factor for emergency deliveries and neonatal morbidity, and therefore was additionally adjusted for in the mediation analysis regarding emergency deliveries.

Because exclusions due to missing data may lead to selection bias, we conducted a sensitivity analysis using the multiple imputation of missing values with chained equations^27^ (missing proportions in the variables ranging from 0.05% to 13.73%; see Table 1 for potential confounders and Table 2 for BMI). Ten imputations with 50 iterations each were implemented.

**Table 1.** Maternal and infant characteristics, overweight- or obesity-related pregnancy complications and rates of asphyxia-related outcomes. Preterm singleton infants without congenital malformation in Sweden 1997–2011

Characteristics	No. of infants (%)	Apgar score 0–3 at 5 minutes	Apgar score 0–3 at 10 minutes	Neonatal seizures	IVH grades 1–4	IVH grades 3–4
		No. of cases (rate)^a^	No. of cases (rate)^a^^,b^	No. of cases (rate)^a^	No. of cases (rate)^a^	No. of cases (rate)^a^
Total	62 499 (100)	766 (12.3)	438 (7.0)	304 (4.9)	895 (14.3)	216 (3.5)
**Maternal characteristics**						
Age at delivery (years)						
<19	1386 (2.2)	28 (20.2)	14 (10.1)	2 (1.4)	23 (16.6)	7 (5.1)
20–24	9128 (14.6)	112 (12.3)	75 (8.2)	41 (4.5)	108 (11.8)	24 (2.6)
25–29	19 240 (30.8)	212 (11.0)	127 (6.6)	79 (4.1)	266 (13.8)	56 (2.9)
30–34	19 881 (31.8)	232 (11.7)	130 (6.5)	114 (5.7)	280 (14.1)	69 (3.5)
≥35	12 864 (20.6)	182 (14.1)	92 (7.2)	68 (5.3)	218 (16.9)	60 (4.7)
Parity						
1	33 869 (54.2)	394 (11.6)	223 (6.6)	155 (4.6)	498 (14.7)	117 (3.5)
2–3	24 550 (39.3)	311 (12.7)	186 (7.6)	126 (5.1)	337 (13.7)	85 (3.5)
≥4	4080 (6.5)	61 (15.0)	29 (7.1)	23 (5.6)	60 (14.7)	14 (3.4)
Cigarette smoking during pregnancy						
No	49 966 (79.9)	546 (10.9)	317 (6.3)	229 (4.6)	638 (12.8)	156 (3.1)
Yes	7368 (11.8)	86 (11.7)	48 (6.5)	36 (4.9)	103 (14.0)	23 (3.1)
Data missing	5165 (8.3)	134 (25.9)	73 (14.2)	39 (7.6)	154 (29.8)	37 (7.2)
Height (cm)						
<155	3006 (4.8)	44 (14.6)	29 (9.6)	14 (4.7)	54 (18.0)	14 (4.7)
155–164	24 649 (39.4)	288 (11.7)	174 (7.1)	103 (4.2)	340 (13.8)	83 (3.4)
165–174	28 714 (45.9)	346 (12.0)	189 (6.6)	152 (5.3)	401 (14.0)	95 (3.3)
≥175	4360 (7.0)	56 (12.8)	34 (7.8)	21 (4.8)	51 (11.7)	10 (2.3)
Data missing	1770 (2.8)	32 (18.1)	12 (6.8)	14 (7.9)	49 (27.7)	14 (7.9)
Education (years)						
≤11	17 792 (28.5)	268 (15.1)	171 (9.6)	97 (5.5)	262 (14.7)	70 (3.9)
12–14	25 120 (40.2)	288 (11.5)	154 (6.1)	118 (4.7)	359 (14.3)	82 (3.3)
≥15	19 044 (30.5)	204 (10.7)	110 (5.8)	88 (4.6)	261 (13.7)	61 (3.2)
Data missing	543 (0.9)	6 (11.0)	3 (5.5)	1 (1.8)	13 (23.9)	3 (5.5)
Country of birth						
Nordic^c^	51 127 (81.8)	577 (11.3)	318 (6.2)	253 (4.9)	710 (13.9)	170 (3.3)
Non-Nordic	11 342 (18.1)	189 (16.7)	119 (10.5)	51 (4.5)	185 (16.3)	46 (4.1)
Data missing	30 (0.0)	0 (0.0)	1 (33.3)	0 (0.0)	0 (0.0)	0 (0.0)
Hypertensive disease						
No	53 537 (85.7)	635 (11.9)	387 (7.2)	256 (4.8)	753 (14.1)	188 (3.5)
Chronic	1090 (1.7)	19 (17.4)	7 (6.4)	5 (4.6)	26 (23.9)	4 (3.7)
Pre-eclampsia	7872 (12.6)	112 (14.2)	44 (5.6)	43 (5.5)	116 (14.7)	24 (3.0)
Diabetic disease						
No	60 011 (96.0)	736 (12.3)	423 (7.1)	291 (4.8)	878 (14.6)	211 (3.5)
Pre-gestational	1368 (2.2)	21 (15.4)	8 (5.8)	9 (6.6)	6 (4.4)	2 (1.5)
Gestational	1120 (1.8)	9 (8.0)	7 (6.3)	4 (3.6)	11 (9.8)	3 (2.7)
Preterm premature rupture of the membranes						
No	45 761 (73.2)	598 (13.1)	325 (7.1)	260 (5.7)	657 (14.4)	168 (3.7)
Yes	16 738 (26.8)	168 (10.0)	113 (6.8)	44 (2.6)	238 (14.2)	48 (2.9)
Year of delivery						
1997–2000	15 166 (24.3)	181 (11.9)	140 (9.2)	73 (4.8)	148 (9.8)	39 (2.6)
2001–2004	16 404 (26.2)	174 (10.6)	100 (6.1)	73 (4.5)	221 (13.5)	61 (3.7)
2005–2008	17 347 (27.8)	211 (12.2)	107 (6.2)	99 (5.7)	293 (16.9)	68 (3.9)
2009–2011	13 582 (21.7)	200 (14.7)	91 (6.7)	59 (4.3)	233 (17.2)	48 (3.5)
**Infant characteristics**						
Gestational age (weeks)						
32–36	54 713 (87.5)	278 (5.1)	169 (3.1)	204 (3.7)	125 (2.3)	19 (0.3)
<32	7786 (12.5)	488 (62.7)	269 (34.7)	100 (12.8)	770 (98.9)	197 (25.3)
Birthweight-for-gestational age (percentile)						
<3	5739 (9.2)	108 (18.8)	45 (7.8)	32 (5.6)	155 (27.0)	36 (6.3)
3 to <10	5054 (8.1)	83 (16.4)	43 (8.5)	31 (6.1)	102 (20.2)	28 (5.5)
10–90	44 379 (71.0)	461 (10.4)	265 (6.0)	194 (4.4)	599 (13.5)	143 (3.2)
90–97	3278 (5.2)	16 (4.9)	13 (4.0)	14 (4.3)	12 (3.7)	3 (0.9)
≥97	3262 (5.2)	43 (13.2)	31 (9.5)	18 (5.5)	11 (3.4)	1 (0.3)
Data missing	787 (1.3)	55 (69.9)	41 (52.2)	15 (19.1)	16 (20.3)	5 (6.4)
Emergency deliveries						
No^d^	50 294 (80.5)	572 (11.4)	344 (6.8)	232 (4.6)	704 (14.0)	168 (3.3)
Yes^e^	12 205 (19.5)	194 (15.9)	94 (7.7)	72 (5.9)	191 (15.6)	48 (3.9)
Sex						
Male	33 594 (53.8)	441 (13.1)	239 (7.1)	179 (5.3)	535 (15.9)	135 (4.0)
Female	28 905 (46.3)	325 (11.2)	199 (6.9)	125 (4.3)	360 (12.5)	81 (2.8)

IVH, intraventricular haemorrhage.

a Rate is calculated as the number of cases per 1000 births.

b Thirty-three infants had missing information on Apgar scores at 10 minutes.

c Nordic countries include: Sweden, Denmark, Finland, Iceland and Norway.

d No emergency deliveries include non-instrumental vaginal delivery (60.0%) and elective caesarean section (20.5%).

e Emergency deliveries include instrumental vaginal delivery (4.4%) and emergency caesarean section (15.1%).

**Table 2.** Association between maternal BMI and severe asphyxia-related outcomes using the conventional and fetuses-at-risk approaches: preterm singleton infants without congenital malformation in Sweden 1997–2011

Outcomes	Per 10 units of BMI^a^	Maternal BMI				
		<25	25 to <30	30 to <35	≥35	Missing
**Conventional approach**						
No. of infants (%)		33 324 (53.3)	13 403 (21.4)	4849 (7.8)	2342 (3.7)	8581 (13.7)
Apgar score 0–3 at 5 minutes						
No. of cases (rate)^b^		341 (10.2)	152 (11.3)	75 (15.5)	42 (17.9)	156 (18.2)
Adjusted RR (95% CI)	1.32 (1.13–1.54)	1 [Reference]	1.08 (0.89–1.31)	1.38 (1.07–1.79)	1.68 (1.21–2.33)	
Apgar score 0–3 at 10 minutes^c^						
No. of cases (rate)^b^		196 (5.9)	90 (6.7)	38 (7.8)	27 (11.5)	87 (10.2)
Adjusted RR (95% CI)	1.37 (1.12–1.67)	1 [Reference]	1.13 (0.88–1.46)	1.24 (0.86–1.78)	1.94 (1.28–2.92)	
Neonatal seizures						
No. of cases (rate)^b^		159 (4.8)	47 (3.5)	27 (5.6)	19 (8.1)	52 (6.1)
Adjusted RR (95% CI)	1.28 (1.00–1.65)	1 [Reference]	0.75 (0.54–1.04)	1.08 (0.70–1.65)	1.66 (1.02–2.68)	
IVH grades 1–4						
No. of cases (rate)^b^		416 (12.5)	177 (13.2)	66 (13.6)	43 (18.4)	193 (22.5)
Adjusted RR (95% CI)	1.18 (1.01–1.37)	1 [Reference]	1.04 (0.87–1.24)	1.01 (0.77–1.33)	1.38 (1.00–1.91)	
**Fetuses-at-risk approach**						
No. of live fetuses (%)		785 173 (57.8)	300 283 (22.1)	94 698 (7.0)	38 039 (2.8)	140 173 (10.3)
Apgar score 0–3 at 5 minutes						
No. of cases (rate)^d^		341 (0.4)	152 (0.5)	75 (0.8)	42 (1.1)	156 (1.1)
Adjusted RR (95% CI)	1.54 (1.30–1.82)	1 [Reference]	1.14 (0.93–1.38)	1.65 (1.27–2.14)	2.38 (1.71–3.33)	
Apgar score 0–3 at 10 minutes^c^						
No. of cases (rate)^d^		196 (0.2)	90 (0.3)	38 (0.4)	27 (0.7)	87 (0.6)
Adjusted RR (95% CI)	1.61 (1.29–2.00)	1 [Reference]	1.19 (0.92–1.53)	1.48 (1.03–2.13)	2.78 (1.83–4.23)	
Neonatal seizures						
No. of cases (rate)^d^		159 (0.2)	47 (0.2)	27 (0.3)	19 (0.5)	52 (0.4)
Adjusted RR (95% CI)	1.48 (1.1–1.95)	1 [Reference]	0.78 (0.56–1.08)	1.27 (0.82–1.96)	2.31 (1.42–3.75)	
IVH grades 1–4						
No. of cases (rate)^d^		416 (0.5)	177 (0.6)	66 (0.7)	43 (1.1)	193 (1.4)
Adjusted RR (95% CI)	1.37 (1.16–1.63)	1 [Reference]	1.09 (0.91–1.31)	1.21 (0.92–1.60)	1.97 (1.42–2.73)	

BMI, body mass index; IVH, intraventricular haemorrhage; RR, risk ratio.

Model adjusted for maternal age at delivery, parity, smoking during pregnancy, height, education, country of birth and year of delivery.

a Ten units is the difference between the median BMIs of mothers with obesity (33.1) and without obesity (23.0).

b Rate in the conventional approach is calculated as the number of cases per 1000 births.

c Thirty-three infants had missing information on Apgar scores at 10 minutes.

d Rate in the fetuses-at-risk approach is calculated as the number of cases per 1000 live fetuses.

Data preparation was performed using SAS version 9.4 (SAS institute Inc., Cary, NC, USA). Statistical analyses were performed using Stata version 15.1 (StataCorp LP, College Station, TX, USA) and SAS (CAUSALMED procedure for mediation analysis).

## Results

Among 62 499 preterm singleton infants born during 1997–2011, there were 766 infants with Apgar score 0–3 at 5 minutes (12.3/1000 births), 438 with Apgar score 0–3 at 10 minutes (7.0/1000 births), 304 with neonatal seizures (4.9/1000 births) and 895 with IVH grades of 1–4 (14.3/1000 births) (Table 1). The mean early-pregnancy BMI was 24.8 (SD, 4.8); 2.8% of the mothers were underweight (BMI < 18.5), 50.6% were of normal weight (BMI 18.5 to <25), 21.4% were overweight (BMI 25 to <30), 7.8% were mildly obese (BMI 30 to <35) and 3.7% were severely obese (BMI ≥35). The prevalence of severe asphyxia-related outcomes according to maternal and infant characteristics and overweight- or obesity-related pregnancy complications are shown in Table 1.

Linear relationships between maternal BMI (per 10-unit increase) and severe neonatal asphyxia-related complications were demonstrated (Table 2). In the conventional analysis, compared with infants of mothers with BMI <25, infants of mothers with BMI ≥35 had increased risks of low Apgar scores at 5 and 10 minutes, neonatal seizures and IVH grades 1–4. When using the FAR approach, corresponding risks were higher (Table 2). BMI 30 to <35 in mothers was also associated with increased risks of low Apgar scores at 5 and 10 minutes. No association was observed between underweight woman (BMI < 18.5) and outcomes (Supplementary Table 3, available as Supplementary data at *IJE* online).

In very preterm infants, no association was shown between maternal BMI and severe asphyxia-related complications using the conventional approach (Table 3). However, when using the FAR approach, higher BMI (per 10-unit increase) was associated with increased risks of low Apgar scores at 5 and 10 minutes and IVH grades of 1–4, but there was no association with neonatal seizures. In moderately preterm infants, maternal BMI ≥35 was associated with increased risks of low Apgar scores at 5 and 10 minutes and neonatal seizures, when using both the conventional and the FAR approach (Table 4); no association was observed between maternal BMI and IVH grades 1–4. For IVH grades of 3–4, neither the conventional nor the FAR approach showed an overall or gestational-age-specific association with maternal BMI (Supplementary Table 4, available as Supplementary data at *IJE* online). In the sensitivity analyses with multiple imputation of missing data, the overall associations between maternal BMI and asphyxia-related complications were essentially unchanged (Supplementary Table 5, available as Supplementary data at *IJE* online).

**Table 3.** Association between maternal BMI and severe asphyxia-related outcomes using the conventional and fetuses-at-risk approaches: very preterm (<32 weeks) singleton infants without congenital malformation in Sweden 1997–2011

Outcomes	Per 10 units of BMI^a^	Maternal BMI			
		<25	25 to <30	30 to <35	≥35
**Conventional approach** ^b^					
Apgar score 0–3 at 5 minutes					
Adjusted RR (95% CI)	1.06 (0.88–1.27)	1 [Reference]	0.93 (0.73–1.19)	1.09 (0.79–1.50)	1.07 (0.72–1.61)
Apgar score 0–3 at 10 minutes^c^					
Adjusted RR (95% CI)	1.05 (0.81–1.36)	1 [Reference]	0.88 (0.6–1.23)	1.04 (0.66–1.63)	1.17 (0.68–2.01)
Neonatal seizures					
Adjusted RR (95% CI)	0.80 (0.47–1.34)	1 [Reference]	0.47 (0.24–0.90)	0.72 (0.33–1.59)	0.71 (0.26–1.95)
IVH grades 1–4					
Adjusted RR (95% CI)	0.97 (0.83–1.14)	1 [Reference]	0.99 (0.82–1.19)	0.89 (0.68–1.17)	0.91 (0.65–1.28)
**Fetuses-at-risk approach** ^d^					
Apgar score 0–3 at 5 minutes					
Adjusted RR (95% CI)	1.57 (1.29–1.93)	1 [Reference]	1.10 (0.85–1.41)	1.63 (1.17–2.28)	2.26 (1.47–3.48)
Apgar score 0–3 at 10 minutes^c^					
Adjusted RR (95% CI)	1.55 (1.17–2.06)	1 [Reference]	1.03 (0.73–1.45)	1.51 (0.95–2.41)	2.47 (1.41–4.31)
Neonatal seizures					
Adjusted RR (95% CI)	1.07 (0.59–1.92)	1 [Reference]	0.52 (0.27–1.01)	0.97 (0.43–2.19)	1.31 (0.47–3.61)
IVH grades 1–4					
Adjusted RR (95% CI)	1.45 (1.21–1.73)	1 [Reference]	1.17 (0.96–1.42)	1.34 (1.00–1.79)	1.92 (1.33–2.76)

BMI, body mass index; IVH, intraventricular haemorrhage; RR, risk ratio.

Model adjusted for maternal age at delivery, parity, smoking during pregnancy, height, education, country of birth and year of delivery.

a Ten units is the difference between the median BMIs of mothers with obesity (33.1) and without obesity (23.0).

b In the conventional approach, rate is calculated as the number of cases per 1000 births.

c Thirty-three infants had missing information on Apgar scores at 10 minutes.

d In the fetuses-at-risk approach, rate is calculated as the number of cases per 1000 live fetuses.

**Table 4.** Association between maternal BMI and severe asphyxia-related outcomes using the conventional and fetuses-at-risk approaches: moderately preterm (32–36 weeks) singleton infants without congenital malformation in Sweden 1997–2011

Outcomes	Per 10 units of BMI^a^	Maternal BMI			
		<25	25 to <30	30 to <35	≥35
**Conventional approach** ^b^					
Apgar score 0–3 at 5 minutes					
Adjusted RR (95% CI)	1.37 (1.07–1.75)	1 [Reference]	1.18 (0.87–1.61)	1.50 (0.99–2.28)	2.01 (1.21–3.35)
Apgar score 0–3 at 10 minutes^c^					
Adjusted RR (95% CI)	1.50 (1.11–2.02)	1 [Reference]	1.38 (0.94–2.03)	1.23 (0.68–2.21)	2.45 (1.32–4.53)
Neonatal seizures					
Adjusted RR (95% CI)	1.46 (1.10–1.93)	1 [Reference]	0.87 (0.59–1.28)	1.20 (0.73–1.99)	2.08 (1.21–3.59)
IVH grades 1–4					
Adjusted RR (95% CI)	0.91 (0.57–1.45)	1 [Reference]	0.71 (0.42–1.18)	0.52 (0.21–1.28)	1.73 (0.83–3.60)
**Fetuses-at-risk approach** ^d^					
Apgar score 0–3 at 5 minutes					
Adjusted RR (95% CI)	1.50 (1.14–1.96)	1 [Reference]	1.20 (0.88–1.64)	1.68 (1.10–2.56)	2.59 (1.55–4.38)
Apgar score 0–3 at 10 minutes^c^					
Adjusted RR (95% CI)	1.70 (1.23–2.37)	1 [Reference]	1.44 (0.98–2.11)	1.43 (0.79–2.60)	3.32 (1.76–6.24)
Neonatal seizures					
Adjusted RR (95% CI)	1.69 (1.24–2.29)	1 [Reference]	0.91 (0.62–1.34)	1.43 (0.86–2.39)	2.87 (1.65–5.01)
IVH grades 1–4					
Adjusted RR (95% CI)	0.97 (0.57–1.63)	1 [Reference]	0.72 (0.43–1.22)	0.59 (0.23–1.47)	2.21 (1.06–4.63)

BMI, body mass index; IVH, intraventricular haemorrhage; RR, risk ratio.

Model adjusted for maternal age at delivery, parity, smoking during pregnancy, height, education, country of birth and year of delivery.

a Ten units is the difference between the median BMIs of mothers with obesity (33.1) and without obesity (23.0).

b In the conventional approach, rate is calculated as the number of cases per 1000 births.

c Thirty-three infants had missing information on Apgar scores at 10 minutes.

d In the fetuses-at-risk approach, rate is calculated as the number of cases per 1000 live fetuses.

In the mediation analyses (Table 5), gestational age <32 weeks mediated 50.9%, 43.1% and 11.2% of the associations between maternal BMI and risks of low Apgar score at 5 and 10 minutes and neonatal seizures, respectively; similar results were found with gestational age as a continuous variable. Maternal hypertensive or diabetic diseases only mediated 8.8% of the association between maternal BMI and low Apgar score at 5 minutes. Emergency deliveries and preterm premature rupture of membranes did not contribute as a mediator for any of the asphyxia-related complications.

**Table 5.** Mediation analysis for the associations between maternal BMI and severe asphyxia-related outcomes: preterm singleton infants without congenital malformation in Sweden 1997–2011 (conventional approach)

Mediators	*P*-value for BMI–mediator interaction	Adjusted RR (95% CI)^a^			Proportion mediated (%)^d^
		Natural direct effect^b^	Natural indirect effect	Total effect^c^	
**Apgar score 0–3 at 5 minutes**					
Maternal hypertensive or diabetic diseases	0.53	1.30 (1.09–1.50)	1.02 (0.99–1.06)	1.33 (1.12–1.53)	8.8
Preterm premature rupture of membranes	0.06	1.32 (1.12–1.53)	1.00 (1.00–1.00)	1.32 (1.12–1.53)	0.1
Gestational age					
<32 weeks	0.10	1.16 (0.98–1.34)	1.14 (1.11–1.18)	1.33 (1.12–1.53)	50.9
As a continuous variable	0.16	1.11 (0.94–1.28)	1.07 (1.06–1.09)	1.19 (1.01–1.38)	42.6
Emergency deliveries^e^	0.11	1.35 (1.14–1.57)	1.00 (1.00–1.00)	1.35 (1.14–1.57)	0.6
**Apgar score 0–3 at 10 minutes**					
Maternal hypertensive or diabetic diseases	0.11	1.41 (1.12–1.70)	0.97 (0.93–1.01)	1.37 (1.09–1.65)	NA^f^
Preterm premature rupture of membranes	0.01	1.37 (1.10–1.65)	1.00 (1.00–1.00)	1.37 (1.10–1.65)	0.1
Gestational age					
<32 weeks	0.08	1.21 (0.97–1.46)	1.13 (1.10–1.17)	1.38 (1.10–1.65)	43.1
As a continuous variable	0.09	1.16 (0.92–1.40)	1.08 (1.06–1.09)	1.25 (0.99–1.50)	35.5
Emergency deliveries^e^	0.10	1.45 (1.14–1.75)	1.00 (1.00–1.00)	1.45 (1.15–1.76)	0.4
**Neonatal seizures**					
Maternal hypertensive or diabetic diseases	0.98	1.29 (0.97–1.61)	0.99 (0.94–1.05)	1.29 (0.97–1.60)	NA^f^
Preterm premature rupture of membranes	0.48	1.28 (0.97–1.59)	1.00 (1.00–1.00)	1.28 (0.97–1.59)	0.2
Gestational age					
<32 weeks	0.046	1.26 (0.95–1.56)	1.03 (1.00–1.05)	1.29 (0.98–1.60)	11.2
As a continuous variable	0.10	1.21 (0.92–1.50)	1.04 (1.03–1.05)	1.25 (0.95–1.56)	17.2
Emergency deliveries^e^	0.80	1.32 (1.00–1.65)	1.00 (1.00–1.00)	1.32 (1.00–1.65)	0.2
**IVH grades 1–4**					
Maternal hypertensive or diabetic diseases	0.23	1.18 (1.01–1.36)	1.00 (0.97–1.03)	1.18 (1.00–1.36)	NA^f^
Preterm premature rupture of membranes	0.41	1.18 (1.00–1.36)	1.00 (1.00–1.00)	1.18 (1.00–1.36)	0.1
Gestational age					
<32 weeks	0.79	0.97 (0.82–1.11)	1.22 (1.17–1.27)	1.18 (0.99–1.36)	NA^f^
As a continuous variable	0.81	0.94 (0.80–1.08)	1.09 (1.07–1.11)	1.02 (0.87–1.18)	NA^f^
Emergency deliveries^e^	0.10	1.19 (1.00–1.37)	1.00 (1.00–1.00)	1.19 (1.00–1.37)	0.1

BMI, body mass index; IVH, intraventricular haemorrhage; NA, not applicable.

a RRs and 95% CIs were estimated from Poisson regression. One model was fitted with each potential mediator. BMI–mediator associations were modelled using multivariable logistic regression. Covariates included maternal age at delivery, parity, smoking during pregnancy, height, education, country of birth and year of delivery. All effects were estimated for a 10-unit BMI difference from a baseline level of 23.0, at the reference category of each covariate. Interaction between exposure and mediator was tested and, if the *p*-value was <0.05, the exposure–mediator interaction term was retained in the mediation analysis.

b Direct effect estimated at mediator level ‘no’.

c Total-effect estimates may vary across models due to differences in the exposure–mediator interactions.

d Sum of the proportion mediated is not necessarily 100% because the mediators could affect or interact with one another and not all mediators may have been considered.

e Model additionally adjusted for birthweight-for-gestational age percentile.

f Proportion mediated cannot be calculated because the natural direct and natural indirect effects were of opposite signs.

## Discussion

### Principal findings

In this nationwide Swedish study of preterm infants, increasing maternal BMI, particularly maternal obesity grades II and III, was associated with increasing risks of low Apgar scores at 5 and 10 minutes, neonatal seizures and IVH grades 1–4, using both the conventional and the FAR approach. These associations varied by gestational age and analytical approach. In very preterm infants, no association between maternal BMI and asphyxia-related complications was observed in the conventional approach as opposed to the FAR approach. In moderately preterm infants, increasing maternal BMI was associated with higher risks of low Apgar scores at 5 and 10 minutes and neonatal seizures using both approaches. Associations between maternal BMI and asphyxia-related outcomes were partly mediated through the consequences of maternal overweight and obesity, particularly lower gestational age (<32 weeks).

### Findings in comparison with other studies

The impact of maternal overweight and obesity in itself, without obesity-related complications, on infant outcomes has been reported in previous studies, including increased risks of severe asphyxia-related complications and excess fetal growth and adiposity in term infants,^4^^,^^28^^,^^29^ and asphyxia-related infant mortality and childhood epilepsy in preterm and term infants.^2^^,^^30^ Previous studies on maternal BMI and neonatal morbidity in preterm infants are relatively few. Khalak *et al.* showed that maternal BMI was associated with a higher risk of delivery-room resuscitation in preterm infants.^31^^,^^32^ Chen *et al.* presented a positive relationship between maternal BMI and low Apgar score but did not consider potential confounding factors.^33^ In the present study, we included, apart from low Apgar scores and neonatal seizures, IVH as an outcome. IVH grade 1–4 is a common short-term morbidity resulting from preterm hemodynamic instability, related to circulatory and respiratory status close after birth, and is also associated with an increased risk of long-term disability.^34–36^ In this study, we contributed to a thorough understanding of the association between maternal BMI and severe asphyxia-related complications in preterm infants, which was partly mediated through lower gestational age.

Mechanisms underlying the associations of maternal overweight and obesity in early pregnancy and adverse consequences for offspring are complex. In early developmental stages (both preconception and periconception), maternal obesity can cause epigenetic modifications, which may influence the normal regulation of brain development, inflammatory responses, immune signalling, glucose and lipid homeostasis, oxidative stress and the commitment and renewal of stem cells.^37^^,^^38^ Placental changes with alterations in nutrient transport, inflammation and lipotoxicity are linked to insulin resistance^37^ and fetal hyperinsulinemia, which may be related to chronic hypoxia even without diabetes.^39^

Our finding that maternal obesity-related risks may differ by analytic approach raises questions about the advantages and disadvantages of the different approaches. Evaluation of the impact of prenatal exposure on post-delivery outcomes selected by gestational age might be subject to potential collider stratification bias.^25^ The FAR approach, which treats gestational age as survival time and respects the fetus–infant continuum,^22^ prevents the collider stratification bias and is therefore preferable for addressing causal questions between prenatal determinants and neonatal-related outcomes.^40^ In contrast, clinicians involved in neonatal units with live-born infants are interested in assessing risks and prognoses at birth based on gestational age and may therefore be more familiar with the conventional approach.^40^ These two approaches present the studied associations from different perspectives, and also contribute as an example of how collider stratification bias could affect and be addressed in perinatal studies.

### Clinical relevance

Several maternal and perinatal factors have been identified to increase the need for neonatal resuscitation.^41^^,^^42^ The management of preterm infants during the first hour of life, namely the ‘Golden hour’, is standardized in international guidelines.^43^^,^^44^ Our findings may be relevant for optimizing preparation and teamwork before delivery, and also add to the evidence to support interventions to reduce obesity in women of reproductive age.

### Strengths and limitations of the present study

To our knowledge, this is the first study to investigate maternal BMI and the risks of asphyxia-related complications in preterm infants. We used two different statistical approaches with adjustment for potential confounders. The population-based design, including a large sample size and prospectively collected data in a standardized healthcare system, limited the risks of selection and information bias.

There are also some limitations. Information on maternal BMI was missing in 13.7% of the study population. Complete case analysis may have led to bias due to missing information on covariates. However, multiple imputation analysis, despite the assumption of missing data at random,^27^ provided similar results. In spite of having data on many confounders, we cannot rule out the possibility of residual confounding by unmeasured or unknown information on maternal factors that may impact the relationship between maternal BMI and asphyxia-related complications in preterm infants. We also lacked specific information on obstetric interventions and neonatal resuscitation efforts. The diagnosis of periventricular leukomalacia concerned only a few preterm infants, which made it impossible to evaluate the impact of maternal BMI on this outcome.

## Conclusion

Increasing maternal BMI, particularly maternal obesity grades II and III, in early pregnancy was associated with an increased risk of low Apgar score at 5 and 10 minutes, neonatal seizures and IVH in preterm infants. These associations were partly mediated through lower gestational age. Preterm deliveries of obese women should be cautiously managed, considering the elevated risk of asphyxia-related neonatal complications.

## Supplementary data

Supplementary data are available at *IJE* online.

## Author contributions

Author contributions Substantial contribution to conception and design: A.M., R.C., S.C.; acquisition of data: S.C.; analysis and interpretation of data: A.M., R.C., S.J., N.R., S.C.; Drafting the article: A.M.; revising it critically for important intellectual content R.C., S.J., N.R., S.C.; Final approval of the version to be published: A.M., R.C., S.J., N.R., S.C.; Agreement to be accountable for all aspects of the work thereby ensuring that questions related to the accuracy or integrity of any part of the work are appropriately investigated and resolved: A.M., R.C., S.J., N.R., S.C.

## Funding

This work was supported by the Swedish Research Council for Health, Working Life, and Welfare (grant 2017–00134) and an unrestricted grant from Karolinska Institutet (Distinguished Professor Award to Professor Cnattingius). No funding bodies had any role in the study design, data collection and analysis, decision to publish or preparation of the manuscript.

## Conflict of interest

None declared.

## Supplementary Material

dyaa088_supplementary_data
