# Supplementary material for: Maternal body mass index in early pregnancy and severe asphyxia-related complications in preterm infants
Source: Int J Epidemiol. 2020 Jun 26;49(5):1647–60. doi: 10.1093/ije/dyaa088 (PMC7746401; doi:10.1093/ije/dyaa088)
Supplement: dyaa088_supplementary_data [file dyaa088_supplementary_data.docx]

**Supplemental information**

| **Supplemental Table 1. ICD-10 codes for maternal and infant diseases** | |
| --- | --- |
| **Diagnoses** | **ICD-10 codes** |
| Major congenital malformation | Q00-Q99, excluding^a^: 17.0 (accessory auricle), Q17.5 (prominent ear), 18.0/Q18.1 (branchial cleft or preaurical sinus), Q25.0 (patent ductus arterious), Q27.0 (absence of umbilical artery), Q31.4/Q32.0 (laryngomalacia), Q38.1 (anchyloglossia), Q52.3 (imperforate hymen), Q53.0-.9 (retentio testis), Q65.0-.9 (congenital hip dislocation), Q66.5-.9 (pes planus), Q69.0/Q69.9 (polydactali), Q70.3 (syndactyly), Q76.0 (spina bifida occulta), Q79.9 (unspecified anomalies of the musculoskeletal system), Q82.5/Q82.9 (congenital nevus) |
| Maternal hypertension | I10-I15, O10, and O11 (chronic hypertension)  O14 and O15 (preeclampsia) |
| Maternal diabetes | E10-E14, and O240-O243 (pregestational diabetes)  O244 (gestational diabetes) |
| Preterm premature rupture of the membranes | O42 |
| ^a^The excluded diagnoses are minor malformations, defined by the Swedish National Board of Health and Welfare (see  https://www.socialstyrelsen.se/globalassets/sharepoint-dokument/dokument-webb/ovrigt/diagnoser-som-inte-ska-rapporteras-om-fosterskador.pdf). | |

**Supplemental Table 2. Test for nonlinear (quadratic) association between maternal BMI and severe asphyxia-related outcomes (conventional approach)**

| **Outcomes** | **Maternal BMI** | |
| --- | --- | --- |
|  | **B (95% CI) for quadratic term** | **p value for quadratic term** |
| **Apgar Score 0-3 at 5 minutes** | 0.000  (-0.002 - 0.002) | 0.99 |
| **Apgar Score 0-3 at 10 minutes** | -0.000  (-0.002 - 0.002) | 0.71 |
| **Neonatal seizures** | 0.001  (-0.001 - 0.003) | 0.19 |
| **IVH grades of 1 to 4** | 0.001  (-0.001 - 0.002) | 0.29 |

BMI=body mass index; IVH= intraventricular haemorrhage

Model adjusted for maternal age at delivery, parity, smoking during pregnancy, height, education, country of birth, and year of delivery.

| **Supplemental Table 3.** Association between maternal BMI, with distinction of underweight woman, and severe asphyxia-related outcomes, using the conventional and fetuses-at-risk approaches. Preterm (<37 weeks) singleton infants without congenital malformation in Sweden 1997-2011 |
| --- |

| **Outcomes** | **Per 10 Units**  **of BMI^a^** | **Maternal BMI** | | | | | |
| --- | --- | --- | --- | --- | --- | --- | --- |
|  |  | **<18.5** | **18.5 to <25** | **25 to <30** | **30 to <35** | **≥35** | **Missing** |
| **Conventional approach** |  |  |  |  |  |  |  |
| No. of infants (%) |  | 1 723 (2.8) | 31 601 (50.6) | 13 403 (21.4) | 4849 (7.8) | 2342 (3.7) | 8581 (13.7) |
| **Apgar Score 0-3 at 5 minutes** | |  |  |  |  |  |  |
| No. of cases (rate)^b^ |  | 12 (7.0) | 329 (10.4) | 152 (11.3) | 75 (15.5) | 42 (17.9) | 156 (18.2) |
| Adjusted RR (95% CI) | 1.32 (1.13-1.54) | 0.60 (0.33-1.10) | 1[Reference] | 1.06 (0.87-1.28) | 1.35 (1.04-1.75) | 1.65 (1.19-2.28) |  |
| **Apgar Score 0-3 at 10 minutes^c^** | |  |  |  |  |  |  |
| No. of cases (rate)^b^ |  | 9 (5.2) | 187 (5.9) | 90 (6.7) | 38 (7.8) | 27 (11.5) | 87 (10.2) |
| Adjusted RR (95% CI) | 1.37 (1.12-1.67) | 0.74 (0.36-1.52) | 1 [Reference] | 1.11 (0.86-1.44) | 1.22 (0.85-1.76) | 1.91 (1.26-2.88) |  |
| **Neonatal seizures** |  |  |  |  |  |  |  |
| No. of cases (rate)^b^ |  | 1 (0.6) | 158 (5.0) | 47 (3.5) | 27 (5.6) | 19 (8.1) | 52 (6.1) |
| Adjusted RR (95% CI) | 1.28 (1.00-1.65) | NA | 1 [Reference] | 0.71 (0.51-0.99) | 1.02 (0.67-1.57) | 1.57 (0.97-2.54) |  |
| **IVH grades of 1 to 4** |  |  |  |  |  |  |  |
| No. of cases (rate)^b^ |  | 13 (7.5) | 403 (12.8) | 177 (13.2) | 66 (13.6) | 43 (18.4) | 193 (22.5) |
| Adjusted RR (95% CI) | 1.18 (1.01-1.37) | 0.59 (0.33-1.04) | 1 [Reference] | 1.01 (0.85-1.22) | 0.99 (0.75-1.30) | 1.35 (0.98-1.87) |  |
| **Fetuses-at-risk approach** | |  |  |  |  |  |  |
| No. of live fetuses (%) |  | 29 613 (2.8) | 755 560 (55.6) | 300 283 (22.1) | 94 698 (7.0) | 38 039 (2.8) | 140 173 (10.3) |
| **Apgar Score 0-3 at 5 minutes** | |  |  |  |  |  |  |
| No. of cases (rate)^d^ |  | 12 (0.4) | 329 (0.4) | 152 (0.5) | 75 (0.8) | 42 (1.1) | 156 (1.1) |
| Adjusted RR (95% CI) | 1.54 (1.30-1.82) | 0.80 (0.44-1.47) | 1 [Reference] | 1.13 (0.93-1.37) | 1.64 (1.26-2.13) | 2.37 (1.70-3.30) |  |
| **Apgar Score 0-3 at 10 minutes^c^** | |  |  |  |  |  |  |
| No. of cases (rate)^d^ |  | 9 (0.3) | 187 (0.2) | 90 (0.3) | 38 (0.4) | 27 (0.7) | 87 (0.6) |
| Adjusted RR (95% CI) | 1.61 (1.29-2.00) | 0.99 (0.48-2.04) | 1 [Reference] | 1.19 (0.92-1.53) | 1.48 (1.02-2.14) | 2.78 (1.83-4.23) |  |
| **Neonatal seizures** |  |  |  |  |  |  |  |
| No. of cases (rate)^d^ |  | 1 (0.0) | 158 (0.2) | 47 (0.2) | 27 (0.3) | 19 (0.5) | 52 (0.4) |
| Adjusted RR (95% CI) | 1.48 (1.13-1.95) | NA | 1 [Reference] | 0.75 (0.54-1.04) | 1.22 (0.79-1.89) | 2.22 (1.37-3.61) |  |
| **IVH grades of 1 to 4** |  |  |  |  |  |  |  |
| No. of cases (rate)^d^ |  | 13 (0.4) | 403 (0.5) | 177 (0.6) | 66 (0.7) | 43 (1.1) | 193 (1.4) |
| Adjusted RR (95% CI) | 1.37 (1.16-1.63) | 0.77 (0.44-1.38) | 1 [Reference] | 1.08 (0.90-1.30) | 1.20 (0.91-1.58) | 1.95 (1.41-2.71) |  |

BMI=body mass index; IVH= intraventricular haemorrhage; RR=Risk ratio; NA= not applicable

Model adjusted for maternal age at delivery, parity, smoking during pregnancy, height, education, country of birth, and year of delivery.

^a^Ten units is the difference in median BMI of mothers with obesity (33.1) and without obesity (23.0)

^b^Rate in the conventional approach is calculated as number of cases per 1000 births

^c^Thirty-three infants had missing information on Apgar scores at 10 minutes

^d^Rate in the fetuses-at-risk approach is calculated as number of cases per 1000 live fetuses

| **Supplemental Table 4.** Association between maternal BMI and intraventricular haemorrhage grades of 3 to 4. Preterm (<37 weeks) singleton infants without congenital malformation in Sweden 1997-2011 | | | | | |
| --- | --- | --- | --- | --- | --- |
| **IVH grades of 3 to 4** | **Maternal BMI** | | | | **Per 10 units**  **of BMI^a^** |
|  | **<25** | **25 to <30** | **30 to <35** | **≥35** |  |
| **Conventional approach^b^** | |  |  |  |  |
| **22-36 gestational weeks** | |  |  |  |  |
| No. of cases (rate) | 107 (3.2) | 40 (3.0) | 12 (2.5) | 12 (5.1) |  |
| Adjusted RR (95% CI) | 1 [Reference] | 0.87  (0.59-1.26) | 0.68  (0.36-1.30) | 1.53  (0.83-2.81) | 1.13  (0.79-1.61) |
| **<32 gestational weeks** | |  |  |  |  |
| No. of cases (rate) | 94 (25.9) | 37 (22.6) | 12 (18.1) | 10 (26.7) |  |
| Adjusted RR (95% CI) | 1 [Reference] | 0.82  (0.55-1.21) | 0.64  (0.34-1.21) | 1.01  (0.52-1.95) | 0.93  (0.64-1.35) |
| **32-36 gestational weeks** | |  |  |  |  |
| No. of cases (rate) | 13 (0.4) | 3 (0.3) | 0 (0.0) | 2 (1.0) |  |
| Adjusted RR (95% CI) | 1 [Reference] | 0.55 (0.16-1.95) | NA | 2.13  (0.48-9.41) | 0.88  (0.33-2.38) |
| **Fetuses–at-risk approach^c^** | |  |  |  |  |
| **22-36 gestational weeks** | |  |  |  |  |
| No. of cases (rate) | 107 (0.1) | 40 (0.1) | 12 (0.1) | 12 (0.3) |  |
| Adjusted RR (95% CI) | 1 [Reference] | 0.92  (0.63-1.34) | 0.83  (0.43-1.58) | 2.21  (1.19-4.09) | 1.33  (0.90-1.96) |
| **<32 gestational weeks** | |  |  |  |  |
| No. of cases (rate) | 94 (0.1) | 37 (0.1) | 12 (0.1) | 10 (0.3) |  |
| Adjusted RR (95% CI) | 1 [Reference] | 0.96  (0.65-1.43) | 0.93  (0.48-1.81) | 2.08  (1.06-4.09) | 1.36  (0.90-2.06) |
| **32-36 gestational weeks** | |  |  |  |  |
| No. of cases (rate) | 13 (0.0) | 3 (0.0) | 0 (0.0) | 2 (0.1) |  |
| Adjusted RR (95% CI) | 1 [Reference] | 0.60  (0.17-2.20) | NA | 3.18  (0.74-13.59) | 1.05  (0.36-3.05) |

BMI=body mass index; IVH=intraventricular haemorrhage; RR=Risk ratio; NA= not applicable

Model adjusted for maternal age at delivery, parity, smoking during pregnancy, height, education, country of birth, and year of delivery.

^a^Ten units is the difference in median BMI of mothers with obesity (33.1) and without obesity (23.0)

^b^In the conventional approach, rate is calculated as number of cases per 1000 births

^c^In the fetuses-at-risk approach, rate is calculated as number of cases per 1000 fetuses

**Supplemental Table 5.** Association between maternal BMI and severe asphyxia-related outcomes. Preterm (<37 weeks) singleton infants without congenital malformation in Sweden 1997-2011 (multiple imputation analysis; Conventional approach)

| **Outcomes** | **Maternal BMI** | | | | **Per 10 Units of BMI^a^** |
| --- | --- | --- | --- | --- | --- |
|  | **<25** | **25 to <30** | **30 to <35** | **≥35** |  |
| **Apgar Score 0-3 at 5 min** | |  |  |  |  |
| Adjusted RR (95%CI) | 1 [Reference] | 1.14  (0.94-1.37) | 1.47  (1.15-1.89) | 1.61  (1.18-2.21) | 1.37  (1.18-1.58) |
| **Apgar Score 0-3 at 10 min^b^** | |  |  |  |  |
| Adjusted RR (95%CI) | 1 [Reference] | 1.20  (0.93-1.54) | 1.34  (0.96-1.87) | 1.80  (1.19-2.74) | 1.43  (1.20-1.70) |
| **Neonatal seizures** |  |  |  |  |  |
| Adjusted RR (95%CI) | 1 [Reference] | 0.75  (0.55-1.03) | 1.05  (0.71-1.57) | 1.44  (0.89-2.33) | 1.25  (0.97-1.62) |
| **IVH grades of 1 to 4** |  |  |  |  |  |
| Adjusted RR (95%CI) | 1 [Reference] | 1.04  (0.86-1.25) | 1.05  (0.81-1.36) | 1.30  (0.92-1.84) | 1.18  (1.03-1.36) |
| **IVH grades of 3 to 4** |  |  |  |  |  |
| Adjusted RR (95%CI) | 1 [Reference] | 0.93  (0.65-1.32) | 0.77  (0.41-1.45) | 1.36  (0.73-2.56) | 1.13  (0.82-1.55) |

BMI=body mass index; IVH=intraventricular haemorrhage; RR=Risk ratio

Model adjusted for maternal age at delivery, parity, smoking during pregnancy, height, education, country of birth, and year of delivery

^a^Ten units is the difference in median BMI of mothers with obesity (33.1) and without obesity (23.0)

^b^Thirty-three infants had missing information on Apgar scores at 10 minutes
